# Prolonged sitting-induced back pain influences abdominal muscle thickness in a sitting but not a supine position

**DOI:** 10.1038/s41598-021-95795-w

**Published:** 2021-08-12

**Authors:** Yeon Kim, Hye-won Kang, Si-hyun Kim, Kyue-nam Park

**Affiliations:** 1 Department of Physical Therapy, College of Medical Science, Jeonju University, 1200 Hyoja-dong, Wansan-gu, Jeonju, Jeollabuk-do 560-759 South Korea; 2 Department of Physical Therapy, Sangji University, Wonju, South Korea

**Keywords:** Signs and symptoms, Pain, Chronic pain

## Abstract

The current study explored whether (i) abdominal muscle thickness differed between non-painful supine and painful sitting positions and (ii) the sitting position was more reliable and useful than the supine position to discriminate between people with and without prolonged sitting-induced lower back pain (LBP). Participants with and without prolonged sitting-induced LBP participated. The thickness of the transversus abdominis (TrA), internal oblique (IO), and external oblique (EO) muscles was measured using ultrasonography in supine, usual sitting, and upright sitting positions. Analysis of variance was used to compare muscle thickness among the positions. Intraclass correlation coefficients and receiver operating characteristic curves were used to determine which position reliably identified between group. The group with LBP showed significantly greater EO muscle thickness than that without LBP only in the upright sitting position. In the group without LBP, the TrA thickness was significantly greater in the usual and upright sitting positions than in the supine position, but there was no significant difference in TrA thickness among three positions in LBP group. Only EO thickness in the upright sitting position significantly predicted prolonged sitting-induced LBP. The current study suggests that clinicians should assess abdominal activation patterns in the upright sitting rather than supine position before applying abdominal muscle motor control training for patients with prolonged sitting-induced LBP, and to distinguish between those with and without prolonged sitting-induced LBP.

## Introduction

In a study of university students, 70.8% complained of lower back pain (LBP) in a sitting position, compared to 23.5% while lying on their back, 3.6% while standing, and 2.6% while lying face down^1^. Prolonged sitting can induce discomfort in the lumbar region and increases the risk of LBP^2^. In cases of LBP after 1 h of sitting, one of the contributing factors might be altered abdominal muscle activation patterns compared to people without a history of LBP^3^. Previous studies comparing participants with and without LBP in a sitting position have suggested that LBP may be associated with changes in the activity of the superficial and deep abdominal muscles^4–7^. A previous study demonstrated that patients who developed LBP after 2 h of prolonged sitting while working on a computer task showed greater electromyographic activity of the external oblique (EO) and internal oblique (IO) muscles compared to those who did not develop LBP^4^.

Ultrasonographic changes in abdominal muscle thickness are considered as an indicator of muscle activation in supine^8–10^. In addition, ultrasonographic measurement provides reliable and valid estimates to quantify abdominal muscle activation and evaluate muscle function^9,11,12^. Ultrasonographic measurements of abdominal muscle thickness of both TrA and IO showed high validation with measures obtained using magnetic resonance imaging and good to high correlation with those obtained using fine-wire electromyography^11,13,14^. An ultrasonographic study found that people without LBP showed greater automatic activation of the transversus abdominis (TrA) in an upright sitting position (hips angled at 90°) than in a supine position, whereas no difference in TrA thickness was found between people with and without LBP in the supine and upright sitting positions^6^. Other ultrasonographic studies also demonstrated no difference in TrA thickness between people with and without LBP during relaxed sitting in a chair with a backrest, in a supine position with a neutral or flexed lumbar area, or in a supine position during a unilateral weight-bearing task^7,8,15^.

Previous studies used a supine position to assess abdominal muscle activation in people with LBP^16,17^. However, these studies failed to demonstrate that the supine position was appropriate for assessing abdominal muscle thickness at rest, to discriminate between people with and without LBP. Thus, further studies are needed to identify the most useful position for such discrimination^7,15^. Assignment of participants to subgroups is important when conducting research on LBP, which is a heterogeneous disorder^18^. For instance, a surface electromyographic study showed no difference in superficial trunk muscle activity between those with and without LBP in the usual sitting position. However, a flexion pattern subgroup showed lower activation of the transverse fibres of IO than the control group^19^.

Clinicians should assess patients in pain-provoking positions to determine whether muscle activation patterns are altered in such positions^20^. Thus, if patients experience LBP after prolonged sitting, clinicians should assess the muscle activation pattern in the sitting, rather that supine or standing, position. However, no study has demonstrated that a sitting position is the most suitable pain-provoking position for assessing abdominal muscle activation patterns of people in whom LBP is provoked by prolonged sitting. Thus, the purpose of the current study was to (i) explore whether, for participants with LBP provoked by prolonged sitting, the thickness of the TrA, EO, and IO muscles differed between the supine (non-painful) and usual and upright sitting (painful) positions, and (ii) determine which position is most reliable for distinguishing between people with and without prolonged sitting-induced LBP.

## Methods

### Participants

We recruited participants after they had finished a 2-h class in Jeonju University and divided them into two groups: a group with LBP provoked by prolonged sitting and a group of healthy controls (Table 1). Participants were included in the LBP group (n = 25) if they (i) experienced LBP after sitting through a 2-h class; (ii) had LBP scores ≥ 30 on a visual analogue scale (VAS) administered during the experiment; (iii) had a history of LBP provoked by prolonged sitting that had lasted for more than 3 months; and (iv) had LBP that was exacerbated by activities involving spinal flexion (e.g. sitting, driving, and forward bending) and relieved by those involving spinal extension (e.g. lying supine, walking, and/or standing)^19,20^. Participants were excluded if they had suspected or diagnosed severe spinal pathology (inflammatory spondyloarthropathy, fracture, or malignancy) or had previously undergone spinal surgery^6^. The pain-free participants (n = 27) had not experienced LBP during the 6 months prior to the study^8^. The VAS LBP pain scores ranged from 0 to 100, with higher scores representing greater pain and disability^21^. The study was carried out in accordance with the Declaration of Helsinki. The participants provided informed written consent, and all procedures were approved by the Jeonju University Institutional Review Board for Human Investigations (jjIRB-180917-HR-2108-0910).

**Table 1:** Subject characteristics.

Characteristics	LBP group (*N* = 25)	Control group (*N* = 27)	P value
Age (years)	21.28 ± 1.28	21.13 ± 1.23	0.44
Height (cm)	164.96 ± 7.88	167.62 ± 11.25	0.14
Weight (kg)	61.64 ± 13.85	63.67 ± 12.72	0.96
VAS (mm)	48.08 ± 10.65	4.65 ± 7.28	< 0.01

Data are expressed as mean ± SD.

*LBP* low back pain, *VAS* visual analogue scale.

### Ultrasound measurement of the abdominal muscle thickness

**Figure 1:**
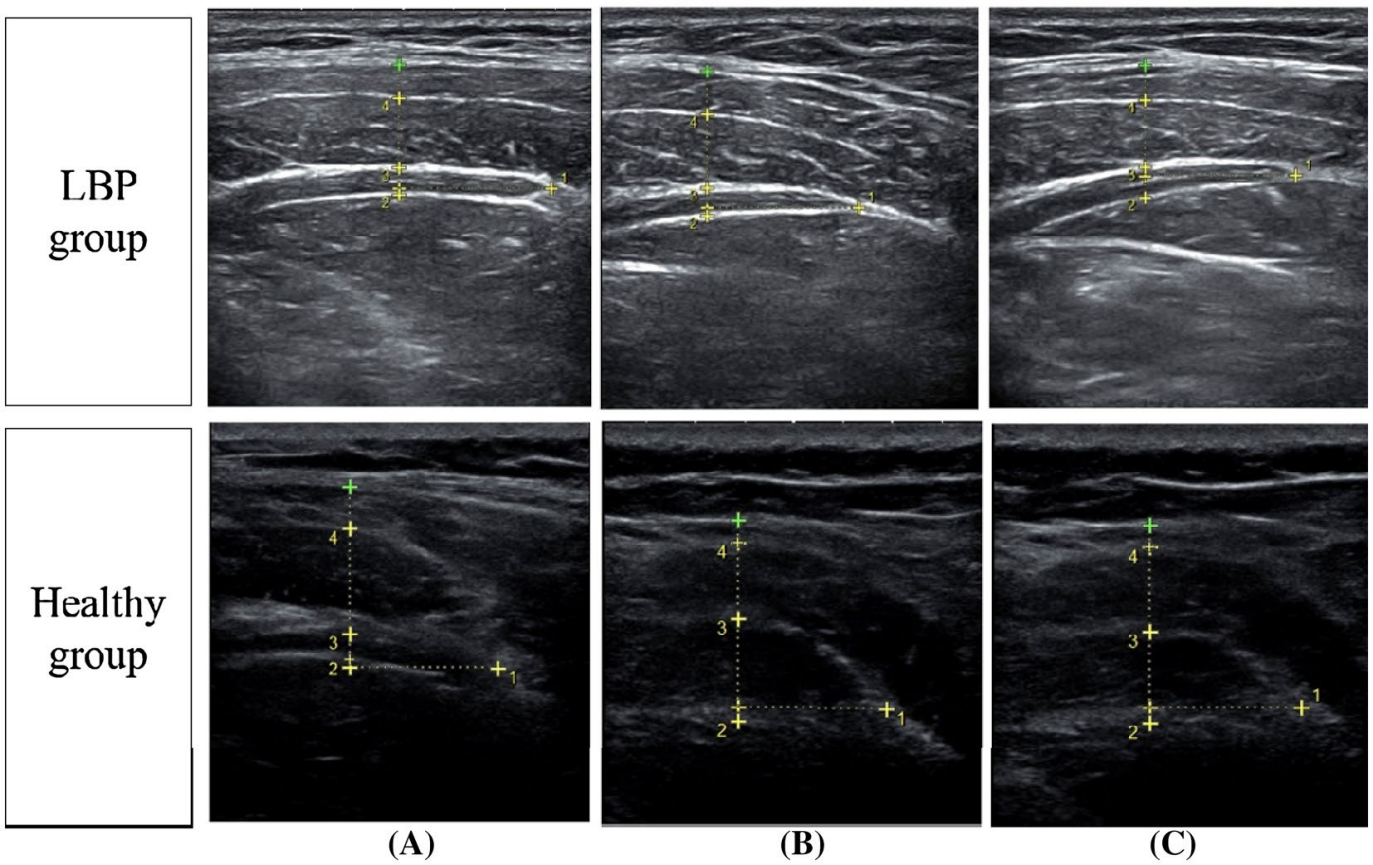
Measurement of the thickness of the transverse abdominis, external oblique, and internal oblique muscles using ultrasonography in the (**A**) supine, (**B**) usual sitting, and (**C**) upright sitting positions, in lower back pain (LBP) group and healthy controls.

Ultrasonographic imaging has been shown to be a reliable and valid technique for assessing muscle function and activity^22^. In this study, ultrasonography (UGEO H60; Samsung Medison Co., Ltd., Korea) with a 38-mm, 13 Hz linear head transducer was used to measure the thickness of the EO, IO, and TrA muscles in the supine, usual sitting, and upright sitting positions. Ultrasound gel was applied between the transducer and skin. The transducer was moved transversely across the right side of the abdominal wall along the participant’s axillary line, midway between the iliac crest and the 12th rib, where a clear image of the lateral abdominal wall muscles and the aponeurotic attachment of the TrA was obtained (Fig. 1)^23^. The participants were advised to breathe naturally; muscle thickness was measured at the end of a relaxed inspiration, because the thickness of the TrA varies with the respiratory cycle^24^. The thickness of the abdominal muscles was measured three times in three different positions at rest, and the average value was used for statistical analysis. The measurements in the supine, usual sitting, and upright sitting positions were carried out in random order, in the same laboratory and by the same examiner.

The participants were instructed to fold their arms across their chest so as not to interfere with the ultrasound measurements. For measurements in the supine position, participants were instructed to lie on the treatment table and look at the ceiling. For measurements in the usual sitting position, the participants were asked to sit comfortably on a backless chair, in the usual sitting position; no further direction as to how to sit were given. For the measurements in the upright sitting position, participants were instructed to sit up straight on a backless chair, with the hips and knees bent to approximately 90°, facing forward with the waist and shoulders in a straight line^25^.

### Data analysis

Muscle thickness on all ultrasonographic images was assessed by a single assessor, who was trained by an experienced specialist for 3 months and was blind to the experimental details, group assignments, and patient records. To measure muscle thickness on frozen ultrasonographic images, cursor points were carefully placed at the inside edge of the fascial band of each muscle, from 2 cm lateral to the V-shaped medial border of the TrA muscle. Muscle thickness was measured in millimetres^26^.

### Statistical analysis

To determine the sample size, we performed a priori power analysis using GPOWER software (version 3.0.10) based on the literature^6^. A previous study indicated that a minimum of 25 participants per group were necessary to detect group differences with a large effect size (Cohen’s d = 0.8), alpha level of 0.05, and power of 0.80^16^. The data were analysed using SPSS software (ver. 26.0; SPSS Inc., USA) by an examiner who was blinded to the group assignments, patients records, and outcomes. The general characteristics of the participants are presented using descriptive statistics. The Kolmogorov–Smirnov test was used to assess the normality of the data. Subjects’ age, height, and weight were compared between the groups with and without LBP using independent *t* tests (Table 1). Two-way random intraclass correlation coefficients (ICCs) [3, 1] were calculated to assess intra-rater reliability for the muscle thickness measurements. The reliability of the measurements was determined using previously reported cut-off scores^27^. Standard error of measurement (SEM) and minimal detectable difference (MDD) values were also calculated.

Separate two-way analyses of variance were used to compare the thickness of abdominal muscles (two groups × three positions). Post hoc Bonferroni correction was applied. The level of significance was set at 0.05 for all statistical analyses.

Receiver operating characteristic (ROC) curves were constructed to investigate the ability of EO, IO, and TrA muscle thickness to discriminate between the LBP and control groups. The area under the ROC curve (AUC) indexes the test’s ability to discriminate between people with and without LBP. An AUC of 1.0 represents perfect discrimination, and an AUC of 0.5 represents discrimination no better than chance^28^.

### Ethics approval

This study approved by the Jeonju University Institutional Review Board for Human Investigations (number: jjIRB-180917-HR-2108-0910).

## Results

Age, height, and weight did not differ between groups (Table 1). The LBP group had higher reported pain levels compared to the control group.

### Intra-rater reliability

The intra-rater reliability for the EO, IO and TrA muscle thickness measurements was excellent in all three positions. The ICC values in the three positions ranged from 0.968 to 0.995. The SEM values were ≤ 0.035, ≤ 0.035, and ≤ 0.048, respectively, for the EO, IO, and TrA muscles. The MDD values ranged from 0.068 to 0.098, 0.070 to 0.097, and 0.058 to 0.133 for the EO, IO, and TrA thickness, respectively (Table 2).

**Table 2:** Intra-rater reliability of measures of abdominal muscle thickness in three positions.

	ICC	95%CI	SEM	MDD
**TrA**				
Supine	0.990	0.982, 0.994	0.021	0.058
Usual sitting	0.990	0.983, 0.994	0.034	0.093
Upright sitting	0.968	0.945, 0.981	0.048	0.133
**IO**				
Supine	0.995	0.990, 0.997	0.025	0.070
Usual sitting	0.994	0.990, 0.997	0.035	0.097
Upright sitting	0.994	0.990, 0.997	0.031	0.086
**EO**				
Supine	0.992	0.986, 0.995	0.025	0.068
Usual sitting	0.992	0.985, 0.995	0.028	0.077
Upright sitting	0.988	0.978, 0.993	0.035	0.098

*ICC* intrarater intraclass coefficient, *CI* confidence interval, *SEM* standard error of measurement, *MDD* minimal detectable difference, *TrA* transverse abdominis muscle, *IO* internal oblique muscle, *EO* external oblique muscle.

### Abdominal muscle thickness

**Figure 2:**
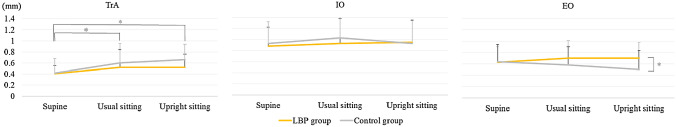
Thickness of the transverse abdominis, external oblique, and internal oblique muscles in the (**A**) supine, (**B**) usual sitting, and (**C**) upright sitting positions, in groups with and without lower back pain.

There was a significant main effect between groups for EO thickness (F = 4.509, P = 0.035). Based on the results of the post hoc analysis, in the upright sitting position, but not in the supine or usual sitting positions, the LBP group showed significantly greater EO muscle thickness than the control group (P = 0.016) (Fig. 2).

There was also a significant main effect within group for the thickness of the TrA muscle (F = 6.129, P = 0.003). In post hoc analysis, the thickness of the TrA muscle in the control group was significantly greater in the usual sitting (P = 0.014) and upright sitting positions (P = 0.001) than in the supine position (Fig. 2). However, the LBP group showed no significant difference in TrA thickness among the three positions. There were no interaction effects (Table 3).

**Table 3:** Comparison of muscle thickness between the low back pain and control groups according to position.

	LBP group			Control group			P value		
	Supine	Usual sitting	Upright sitting	Supine	Usual sitting	Upright sitting	Group	Position	Group × position
TrA	0.41 ± 0.14	0.52 ± 0.32	0.52 ± 0.24	0.42 ± 0.26	0.60 ± 0.35	0.66 ± 0.28	0.070	0.003*	0.449
IO	0.88 ± 0.34	0.93 ± 0.45	0.95 ± 0.40	0.93 ± 0.39	1.03 ± 0.46	0.93 ± 0.41	0.485	0.648	0.765
EO	0.63 ± 0.27	0.70 ± 0.31	0.70 ± 0.27	0.64 ± 0.30	0.58 ± 0.32	0.50 ± 0.34	0.035*	0.748	0.182

Data are expressed as mean ± SD.

*LBP* low back pain, *TrA* transverse abdominis muscle, *IO* internal oblique muscle, *EO* external oblique muscle.

*Means that P value is under 0.05.

### Ultrasound characteristics

Measurement of EO thickness in the upright sitting position was able to distinguish between with and participants without prolonged sitting-induced LBP. The AUC was 0.723 (95% CI = 0.585, 0.861; P = 0.006) for EO thickness in the upright position. EO muscle thickness in the upright position had a sensitivity of 0.600 and specificity of 0.607, for a cut-off value of 0.565 (Table 4).

**Table 4:** ROC analysis of abdominal muscle thickness.

		AUC value	95% CI	P value	Cut-off (mm)	Sensitivity	Specificity
**TrA**							
Supine		0.564	0.406, 0.722	0.430	0.370	0.520	0.536
Usual sitting		0.408	0.251, 0.565	0.255	0.415	0.400	0.429
Upright sitting		0.343	0.191, 0.495	0.053	0.550	0.360	0.357
**IO**							
Supine		0.476	0.317, 0.635	0.769	0.855	0.520	0.500
Usual sitting		0.430	0.272, 0.588	0.388	0.825	0.440	0.439
Upright sitting		0.512	0.351, 0.673	0.883	0.845	0.520	0.536
**EO**							
Supine		0.504	0.343, 0.665	0.963	0.545	0.560	0.536
Usual sitting		0.613	0.460, 0.766	0.163	0.610	0.560	0.526
Upright sitting		0.723	0.585, 0.861	0.006*	0.565	0.600	0.607

*AUC* area under curve, *CI* confidence interval, *TrA* transverse abdominis muscle, *IO* internal obliqu, *EO* external oblique.

*Means that P value is under 0.05.

## Discussion

This study of participants with LBP provoked by prolonged sitting provides evidence that such individuals differ from healthy controls with respect to how the thickness of the superficial and deep abdominal muscles varies to maintain specific positions; moreover, the difference is sufficient to serve as a useful diagnostic tool. The differences in muscle thickness were notable in three main respects: (i) the LBP group showed greater EO thickness than healthy controls only in upright sitting, not in the non-painful supine position; (ii) measurement of EO thickness in the upright sitting position can reliably distinguish between individuals with and without LBP; and (iii) assessment of TrA activation motor control using ultrasonography should be performed in an upright sitting rather than supine position, especially in individuals with LBP provoked by prolonged sitting.

A previous study reported that individuals who developed transient LBP over a 2-h period of sitting showed greater electromyographic activation, ranging from 1.48 to 2.14%, of superficial muscles (EO, IO, and rectus abdominis), whereas individuals who did not develop LBP showed < 1% muscle activation, indicating near-complete relaxation of the superficial muscles^4^. In another ultrasonographic study, the LBP group showed the opposite pattern, with EO muscle activity being dominant in standing tasks, while in the healthy group TrA muscle activity was dominant^5^. In agreement with previous findings, our results showed that only the EO muscle got thicker, by about 1.4 times, in the LBP group relative to pain-free controls, and only while performing the upright sitting position. Taken together with these previous findings, our results suggest that those with LBP provoked by prolonged sitting show a bias toward posture-specific activation of the superficial EO muscle.

The increased thickness of the superficial EO muscle in the LBP group, seen only in the upright sitting position, is likely a consequence of LBP in the sitting position. Our participants reported current and past LBP, however, they did not experience LBP in the supine position. When patients with LBP are instructed to perform certain activities and postures, they may experience pain; altered muscle activation patterns may ensue, particularly reduced activity of the deep trunk muscles and increased activity of the large superficial trunk muscles^29^. Previous experimental studies also demonstrated increased activation of the EO in individuals with LBP when pain was anticipated during postural tasks^30,31^. Thus, the EO muscle might be automatically tuned toward increased activity in the pain-provoking upright sitting position, possibly as a strategy to protect against existing LBP in the upright sitting position.

Whether TrA thickness can discriminate between individuals with and without LBP is controversial. While one previous study reported that the TrA contraction ratio (TrA thickness when contracted relative to that at rest) during abdominal hollowing in the supine (AUC = 0.693) and upright sitting positions (AUC = 0.686) was able to distinguish between those with and without a history of LBP^32^, another study did not^33^. People with LBP have limited opportunity to develop a strategy for contracting the TrA through feedback sessions, so examiners typically have difficulty measuring TrA thickness during voluntary contraction. Additionally, examiners may be unable to repeat the training several times before recording the TrA thickness, due to potential learning effects^32^. Although the current study measured TrA thickness during involuntary contraction due to the difficulties associated with measuring it during voluntary contractions, we did not find TrA thickness measurements useful for discriminating between individuals with and without LBP provoked by prolonged sitting. However, we found a significant group difference in EO thickness during involuntary contraction. The measurement of EO thickness in the upright sitting position proved the most useful (AUC = 0.723, cut-off value = 0.565 cm) for distinguishing between people with and without existing LBP provoked by prolonged sitting. In addition, the intra-rater reliability was high. Therefore, we suggest that measurement of EO thickness in an upright sitting position is a reliable method for identifying people with LBP provoked by prolonged sitting, and also has potential as a tool for motor control training focusing on EO inhibition.

With regard to changes in deep muscle thickness, our healthy control group showed an increase in TrA thickness from the supine to the sitting positions, whereas the LBP group showed such no change even though greater TrA activation would be needed in the sitting than supine position, suggesting motor control dysfunction. In line with our results, previous ultrasonographic studies found that people without LBP showed more TrA activation in the erect sitting posture (hips at 90°) than in slouched sitting and supine positions, whereas people with LBP showed no difference in TrA activation among the supine, slouched, and upright sitting positions^6,24^. Taken together, the previous and current results indicate that clinicians should train patients with LBP provoked by prolonged sitting in involuntary TrA activation in both sitting and supine positions.

The results of this study may help clinicians to understand how the testing position influences muscle thickness on ultrasonography when classifying patients who have LBP during spinal flexion activities, including prolonged sitting, which is relieved by activities involving spinal extension. This study also provides data that could facilitate the planning of rehabilitation programs incorporating motor control exercises for patients with prolonged sitting-induced LBP. A previous study recommended that motor control exercises be performed in a functional position as early as possible, to “re-educate” feedforward mechanisms, and should progress from a position providing greater support to a functional position^34^. O’Sullivan also highlighted the importance of training patients in pain-provoking positions, unless the patient is unable to activate the target muscle in positions providing greater support, such as the supine position^20^. For example, the reduction in EO thickness during selective TrA activation in a standing position rather than in crook lying could be beneficial for rehabilitating LBP patients showing excessive EO activity during selective TrA activation^35^. Therefore, we suggest that a motor control test conducted in an upright sitting rather than supine position could be useful to assess and improve motor control (e.g. EO inhibition and TrA facilitation). In addition, if motor control training were completed in the supine position to promote EO inhibition and TrA activation early in rehabilitation, the training should progress to an upright sitting position for patients with prolonged sitting-induced LBP.

One of the limitations of this study was that only young participants were recruited, so the results cannot be generalised to older populations with LBP. Another limitation was that a preliminary, cross-sectional design was used, so we could not determine cause-and-effect relationships, i.e. whether increased EO and decreased TrA in the upright siting position caused prolonged sitting-induced LBP. Thus, it would be useful for further studies to evaluate the long-term effects of progressive feedback training, focused on inhibition of EO and facilitation of TrA in an upright sitting position, on the ability of patients with prolonged sitting-induced LBP to control these muscles; increasing the duration of sitting without LBP and reducing LBP intensity in the sitting position could be additional goals.

## Conclusion

The current study revealed increased EO thickness in an upright sitting position in participants with prolonged sitting-induced LBP compared to those who did not have LBP. In addition, there were no differences in TrA thickness within the LBP group among positions. These preliminary results indicate that prolonged sitting-induced LBP can influence EO and TrA activation patterns during upright sitting, which is a pain-provoking position, but not in the non-painful supine position. These findings suggest that clinicians should place patients in an upright sitting, rather than supine, position both to assess involuntary activation of the abdominal muscles before applying motor control training of the EO and TrA, and to discriminate between patients with and without prolonged sitting-induced LBP.
